# Impact on Infants’ Cognitive Development of Antenatal Exposure to Iron Deficiency Disorder and Common Mental Disorders

**DOI:** 10.1371/journal.pone.0074876

**Published:** 2013-09-23

**Authors:** Thach Duc Tran, Beverley-Ann Biggs, Tuan Tran, Julie Anne Simpson, Sarah Hanieh, Terence Dwyer, Jane Fisher

**Affiliations:** 1 Research and Training Centre for Community Development, Hanoi, Vietnam; 2 Centre for Women’s Health Gender and Society, Melbourne School of Population and Global Health, the University of Melbourne, Melbourne, Australia; 3 Jean Hailes Research Unit, School of Public Health and Preventive Medicine, Monash University, Melbourne, Australia; 4 Department of Medicine (RMH/WH), the University of Melbourne, the Royal Melbourne Hospital, Melbourne, Australia; 5 Centre for Molecular, Environmental, Genetic & Analytic Epidemiology, Melbourne School of Population and Global Health, the University of Melbourne, Melbourne, Australia; 6 Murdoch Children’s Research Institute, Royal Children’s Hospital, Melbourne, Australia; Aga Khan University, Pakistan

## Abstract

**Objectives:**

The aim of this study was to examine the effects of antenatal exposure to iron deficiency anemia (IDA) and common mental disorders (CMD) on cognitive development of 6 months old infants in a developing country.

**Methods:**

A prospective population-based study in a rural province in Vietnam, which enrolled pregnant women at 12–20 weeks gestation and followed them up with their infants until six months postpartum. Criteria for IDA were Hb <11 g/dL and serum ferritin <15 ng/mL. CMD symptoms were assessed by the Edinburgh Postnatal Depression Scale-Vietnam validation. Infant cognitive development was assessed by Bayley Scales of Infant and Toddler Development, 3^rd^ Ed. Path analyses were performed to determine the direct and indirect, partly or fully mediated, causal effects of the antenatal exposures.

**Results:**

A total of 497 pregnant women were recruited, of those 378 women provided complete data which were included in the analyses. Statistically significant direct adverse effects of persistent antenatal IDA (estimated difference of −11.62 points; 95% CI −23.01 to −0.22) and antenatal CMD (−4.80 points; 95% CI: −9.40 to −0.20) on infant Bayley cognitive scores at six months were found. Higher birthweight, household wealth, and self-rated sufficient supply of breastmilk were associated with higher cognitive scores. Maternal age >30 years and primiparity had an indirect adverse effect on infants’ Bayley cognitive scores.

**Conclusions:**

These findings suggest that antenatal IDA and CMD both have adverse effects on child cognitive development, which if unrecognized and unaddressed are likely to be lasting. It is crucial that both these risks are considered by policy makers, clinicians, and researchers seeking to improve child cognitive function in developing countries.

## Introduction

It is estimated that 200 million children aged less than 5 years in the world do not reach their developmental potential and most of them are living in low- and lower-middle-income countries [1]. The intrauterine environment appears to be a critical determinant of the development of the baby in later life through “programming mechanisms” [2]. Fetal programming postulates that fetal adjustments to adverse conditions *in utero* may cause long-lasting changes in physiological functions that render the brain or body vulnerable to developmental delay and/or illnesses later in life [2]. There is convincing evidence that fetal programming contributes to chronic adult conditions including obesity and cardiovascular diseases [3], . Recently attention has turned to examining fetal programming hypotheses in relation to neurobehavioral outcomes.

Iron deficiency, which at its most severe is termed iron deficiency anemia (IDA), is the most common micronutrient deficiency worldwide [5]. It is estimated that 42% of pregnant women are anemic in low- and lower-middle-income countries [6]. Several longitudinal studies in humans have concluded that foetal or neonatal IDA is associated with behavioral difficulties in infants and children. These include diminished general autonomic response, motor maturity and self-regulation [7], higher levels of negative emotionality and lower levels of alertness and soothability in infants [8], slower neuronal conduction [9], worse learning ability and memory at 3 to 4 years [10], and poorer performance on mental and psychomotor evaluations at preschool age [11]. On the other hand, Rioux et al. [12] in a study of 63 mother infant pairs in Canada found no significant relationship between maternal antenatal iron level and infant’s cognitive performance at 6 months. Several trials of universal antenatal iron supplementation have also concluded that it had no consistent effect on the intelligence quotient of children when they were four years old [13] and child behavior at early school age [14]. All of the human studies were conducted in well-nourished populations in high-income countries.

Beside iron deficiency, maternal mental health status may play a crucial role in fetal and infant development. The common mental disorders (CMD) of depression and anxiety are estimated to occur on average in 10% of pregnant women in high-income countries and 16% (range from 5.2 to 32.9%) in low- and lower-middle-income countries [15]. Studies from high-income countries suggest that antenatal anxiety increases the risk of child behavioral/emotional problems at four years of age [16]; and has a negative association with cognitive ability [17], [18]. However, DiPietro et al. [19] found that antenatal anxiety and depression were associated with higher Bayley II Mental Development Index scores in children aged 24 months. There have been very few attempts to examine this relationship in developing countries where common mental disorders are more prevalent and can interact with other determinants such as micronutrient deficiencies which are common in these settings but not in developed countries. We could find only one published study, which reported no association between symptoms of common mental disorders in mothers in the third trimester of pregnancy and infant cognitive development at 12 months of age [20].

Vietnam is ranked by the World Bank as a lower-middle-income country having only recently been re-classified from being a low-income country. About 30% of women living in rural Vietnam experience CMD during pregnancy or after giving birth [21]. Iron deficiency is the most common micronutrient deficiency and anemia affects about 36.5% pregnant women in this setting [22].

The aim of this study was to test the hypothesis that maternal IDA and clinically significant symptoms of CMD during pregnancy would have adverse effects on the cognitive development of infants at 6 months of age in rural Vietnam.

**Figure 1:**
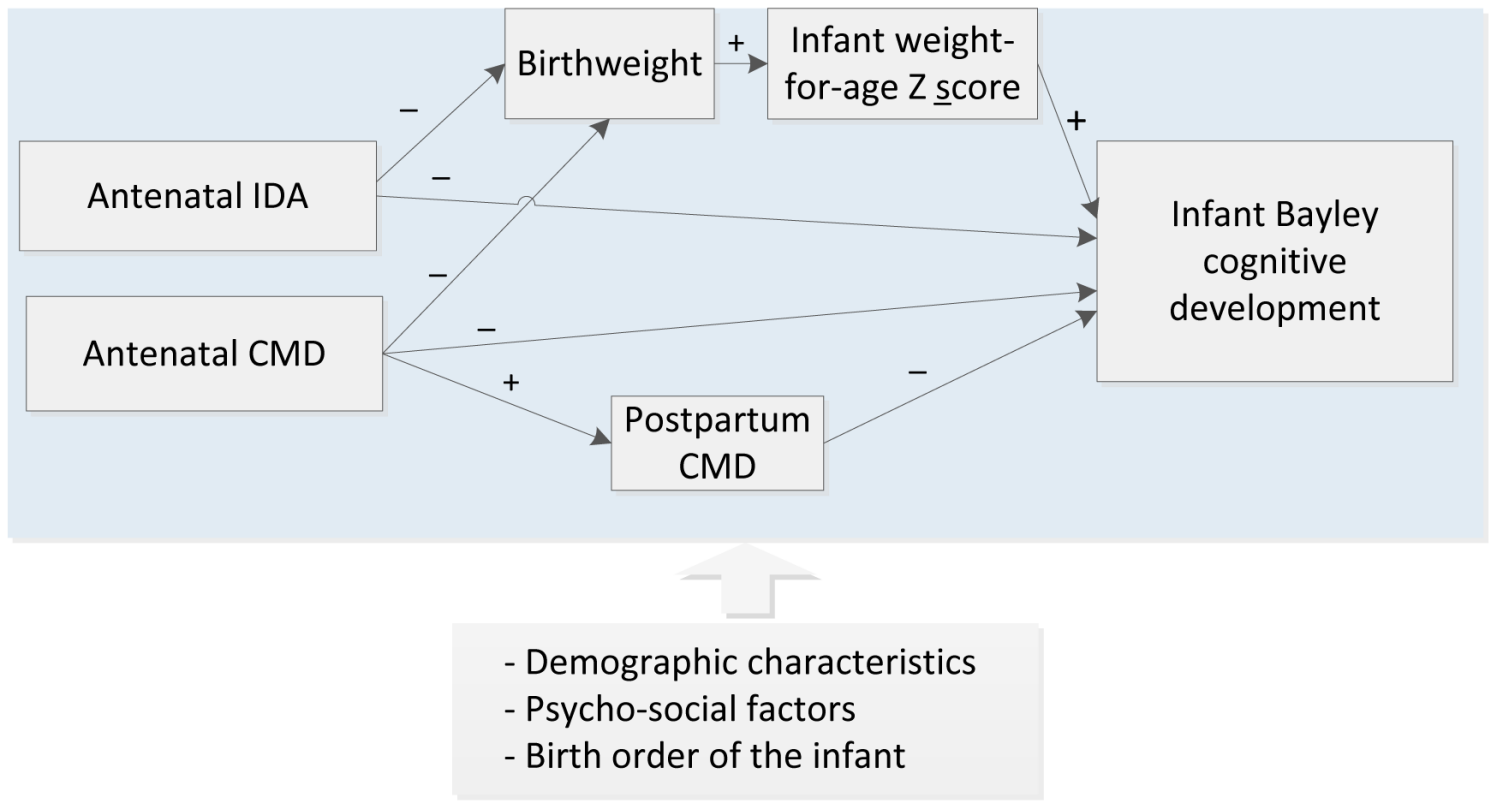
Conceptual model of the direct and indirect effects of antenatal iron deficiency anemia (IDA) and common mental disorders (CMD) on infant cognitive development. Single-headed arrows represent hypothesized directional paths.

The hypothesized model (Figure 1) was constructed from international and Vietnamese evidence. In the model, we postulated that maternal antenatal IDA and/or CMD would affect infant cognitive development at six months via both direct and indirect pathways. Demographic characteristics and other psychosocial factors are potential confounders of both the exposures and the main outcome and therefore had to be included in every aspect of the hypothesized model.

The hypothesized direct pathway was that antenatal IDA and/or CMD symptoms could cause adverse conditions *in utero* which affect fetal neurodevelopment and lead to lasting changes in cognitive function. The hypothesized indirect pathways were first that maternal IDA and/or CMD during pregnancy have adverse effects on birthweight and infant weight and second that maternal postpartum CMD, which is predicted by antenatal CMD, compromises caregiving and that these mediate effects on infant cognitive development. Birthweight and postnatal CMD were included in this model as the main mediators due to their associations with the exposures of interest [23], [24] and the outcome [25] in low and low-middle-income countries.

## Methods

### Study Setting

This study was conducted in Ha Nam, a typical Red River delta rural province in the north of Vietnam located approximately 50 km south of Hanoi. Ha Nam Province has a population of 0.8 million inhabitants. In 2011 the average annual per capita income was approximately USD800 and 7.5% of people lived below the international poverty line of USD1.25 per day. Most births in Ha Nam are at either commune health centers, or at district and provincial hospitals and more than 99% of women attend for antenatal care. Mental health is not considered either in antenatal care or in primary healthcare. The national iron supplementation program was implemented in 1997 and recommends that women take daily iron and folic acid. At the time of this study neither iron supplements nor iodized salt was available for free and women living in the study site were expected to purchase them over-the-counter.

### Study Design

The study was a prospective population-based study in which women were enrolled during pregnancy and followed, with their babies until six months postpartum.

Data were collected in four surveys between December 2009 and March 2011. The first (Wave One, W1) was conducted when the women were recruited before mid-pregnancy and the second (Wave Two, W2) when participants were at least 28 weeks gestation. After childbirth two assessments of mothers and infants were conducted when the babies were 8 weeks (Wave Three, W3) and 6 months (Wave Four, W4) old.

### Participants

Participants were recruited by a two-stage sampling procedure in which first, 50 of the 116 communes in the province were selected randomly by an independent statistician and second all women who were pregnant with a single fetus and between 12 and 20 weeks of gestation in the selected communes during the enrollment period (December 2009 to January 2010) were eligible and invited to participate [26].

### Data Sources

#### Infant cognitive development

Infant cognitive development (collected only at W4) was assessed by directly testing the infant using the Bayley Scales of Infant and Toddler Development 3^rd^ Ed, Cognitive Scale (BSID) [27]. In the original validation studies conducted in US, the reliability coefficient of BSID for infants at 6 months was 0.87 and the stability coefficient was 0.79 [28]. There is current no data available on the validation of the BSID for use in Vietnam.

#### Psycho-social data

Maternal common mental disorders symptoms (CMD, all 4 Ws) were assessed by the Edinburgh Postnatal Depression Scale-Vietnam Validation (EPDS-V) [29], [30]. The EPDS-V includes ten fixed choice items scored from 0–3 which assess dimensions of low mood over the previous seven days and yield a total score from 0 to 30. The EPDS-V was translated from the English version, culturally verified and validated against psychiatrist-administered Structural Clinical Interviews for DSM IV diagnoses to establish local cut off scores for pregnant women and those who have recently given birth. In this setting, where emotional literacy is low, the cut-off point to detect clinically significant symptoms with a sensitivity of 70% and a specificity of 73% is ≥4 [30].

Intimate partner physical, sexual, and emotional abuse and history of child abuse (W1, W2, W4) were assessed by the pregnancy section of the WHO Multi-country study on Domestic Violence survey [31]. This questionnaire has been demonstrated to be appropriate for use in equivalent settings [32].

Social circumstances and reproductive health (W1, W2, W3, and W4) included current non-economic coincidental life adversity; quality of relationship with her mother and mother-in-law; family support in caring for the baby during the day and, at night, and with housework; reproductive health history including gravidity, parity, history of spontaneous abortions, fetal or neonatal deaths; and whether or not the pregnancy was welcome were collected by study-specific questions which have been used in previous studies in the same settings [21]. These questions have been found to be comprehensible to participants and to yield interpretable data.

Household economic status (W1) was assessed by the World Bank method which calculates a Household Wealth Index from information about 17 household characteristics, services and durable assets [33]. Maternal age, marital status, educational level, and occupational status and security of income were collected by study-specific questions [21].

Study-specific structured questions (W3 and W4) were used to assess whether or not a woman was offering any breastfeeds to the infant and her appraisal of whether the amount of milk was sufficient to meet her infant’s needs.

#### Biological data

Maternal hemoglobin (W1, W2) was evaluated in the field from finger stick blood, using a hemoglobinometer (HemoCue AB, Angelholm Sweden). A 3 mL sample of venous blood (W1, W2) was taken from a pregnant woman and centrifuged to harvest serum, frozen at −80°C. Serum ferritin was evaluated by Chemiluminescent Microparticle Immuno Assay performed on the Archicentre ci62000 instrument (Abbott, Illinois, USA) at Alfred Pathology Services, Alfred Health, Australia.

A spot urine sample (W2) was obtained, frozen in a field freezer and transported in a cold chain to the laboratory of the National Hospital of Endocrinology in Hanoi. At the laboratory, urine iodine concentration was determined by means of the Sandell-Kolthoff reaction, as recommended by WHO, UNICEF and the ICCIDD [34].

Infant birthweights were collected by maternal reports at W3. If the mother did not remember or was not sure, the infant birthweight was collected from or verified against the birth records at the health facility. Infant weight (W3, W4) was measured by the Seca 876 Scale which first measures maternal weight and second measures the weight of the infant when held in her arms.

### Procedure

The BSID were translated from English into Vietnamese and back-translated to English for verification [35]. BSID administrators were psychologists practicing at the *TuNa Clinic* which is a service associated with the Research and Training Centre for Community Development, who were experienced in early child development assessment. The administrators were trained by a local expert in BSID and successfully completed a post-training test. This study used the original toolkits and followed the guidelines of the Administration Manual and the training DVDs strictly [36].

A pilot study was conducted to check the language and to standardize the test procedure of BSID and psychosocial questionnaires before conducting this study. Psychosocial data were collected by professional interviewers of the data collection team of the Research and Training Centre for Community Development. The interviewers completed a three-day training course, which included an introduction to the study, detailed discussion on the questionnaires, and a practice in the real-world situation.

As Vietnamese women living in rural areas are unfamiliar with the completion of self-report questionnaires, all data were collected by face-to-face and paper-based interviews. All interviews of mother including administration of BSID were conducted in private rooms at commune health centers.

### Data Management and Data Analysis

Data management and descriptive analyses were performed in STATA version 11 (StataCorp LP, College Station, Texas, USA).

The total raw scores of BSID were converted to composite scores based on the guidelines of Bayley 3^rd^ Edition Manual [36]. In the reference population, composite scores have a range of 55 to 145, a mean of 100, and a standard deviation of 15. This transformation allows for a standardized measurement, which represents in standard deviation units how far an infant’s score is from the mean or average score for infants of that precise age. Composite scores were used in all analyses. Infant’s weight-for-age Z scores (WAZ) were calculated from infant’s age, sex, and weight by WHO Anthro version 3.2.2 (World Health Organization, January 2011). The two exposures of interest, IDA and CMD, were dichotomized into those with and without clinically significant symptoms. Criteria for IDA were Hb <11 g/dL and serum ferritin <15 ng/mL as recommended by the WHO and the United Nations Children’s Fund [37]. Persistent antenatal IDA was defined as meeting the criteria for IDA in both W1 and W2. The EPDS-V was validated as a screening tool which could only classify perinatal women into two groups, those with normal mood and those with probable CMD (cut-off ≥4, [30]). In this setting higher EPDS-V scores have not been shown definitively to be indicative of more severe CMD. A family support variable was derived by summing three variables: having support in caring for the infant during the day time (1: yes; 0; no), during the night (1: yes; 0; no), and assistance with housework (1: yes; 0; no), to yield scores ranging from 0 (no support) to 3 (having all of the three kinds of support). The household’s economic situation was assessed by a wealth index, which is a proxy measure constructed from 17 household characteristics, services and durable assets [33]. The lowest income group was the lowest quartile of the index and the highest income was the highest quartile.

Data were described by frequencies and percentages (for categorical variables); means and standard deviations (for normally distributed continuous variables), and medians and interquartile ranges (for skewed distributions). The direct and indirect effects on infant BSID scores were examined simultaneously by Path Analysis performed in Mplus version 6 (Muthén & Muthén, Los Angeles, United States of America). Other potential contributing factors to the outcome and the mediators were added into the model based on the results of univariate analyses and prior evidence to control for possible confounding. The command “MODINDICES” in Mplus was used after the model estimation to check if there was any significant missing path in the model.

The model was estimated using weighted least-squares and the probit link function that are robust to non-normality. Only one model was fitted which included 22 manifest variables. There were no latent variables in the model. All of the variables were retained in the model even if non-significant. Data of all four waves were used in this analysis. Variables which were collected in multiple waves such as CMD were treated as separate variables and put into the model in the order in which they had been collected. No multilevel technique was used in this analysis.

In order to evaluate model fit, we used the Chi-Square Test of Model Fit with p values greater than 0.05 indicating a good fit, Root Mean Square Error Of Approximation (RMSEA) with values less than 0.05 indicating a good fit, and Tucker-Lewis Index (TLI) and Comparative Fit Index (CFI) with values greater than 0.95 indicating a good fit [38].

### Ethics Approvals

Approvals to conduct the study were provided by the Ha Nam Provincial Health Department Ethics Committee, the Vietnam Medical Association Ethics and Scientific Committee and the University of Melbourne’s Health Sciences Human Ethics Committee. All participants were provided with an oral and written plain language description of the study and either signed a consent form, or those who could not write provided a thumbprint or verbal consent witnessed by an independent observer. The consent procedure was approved by the ethics committees. Permission to transport biological samples from Vietnam to Australia for analyses was provided by the Ministry of Health.

## Results

### Sample

**Figure 2:**
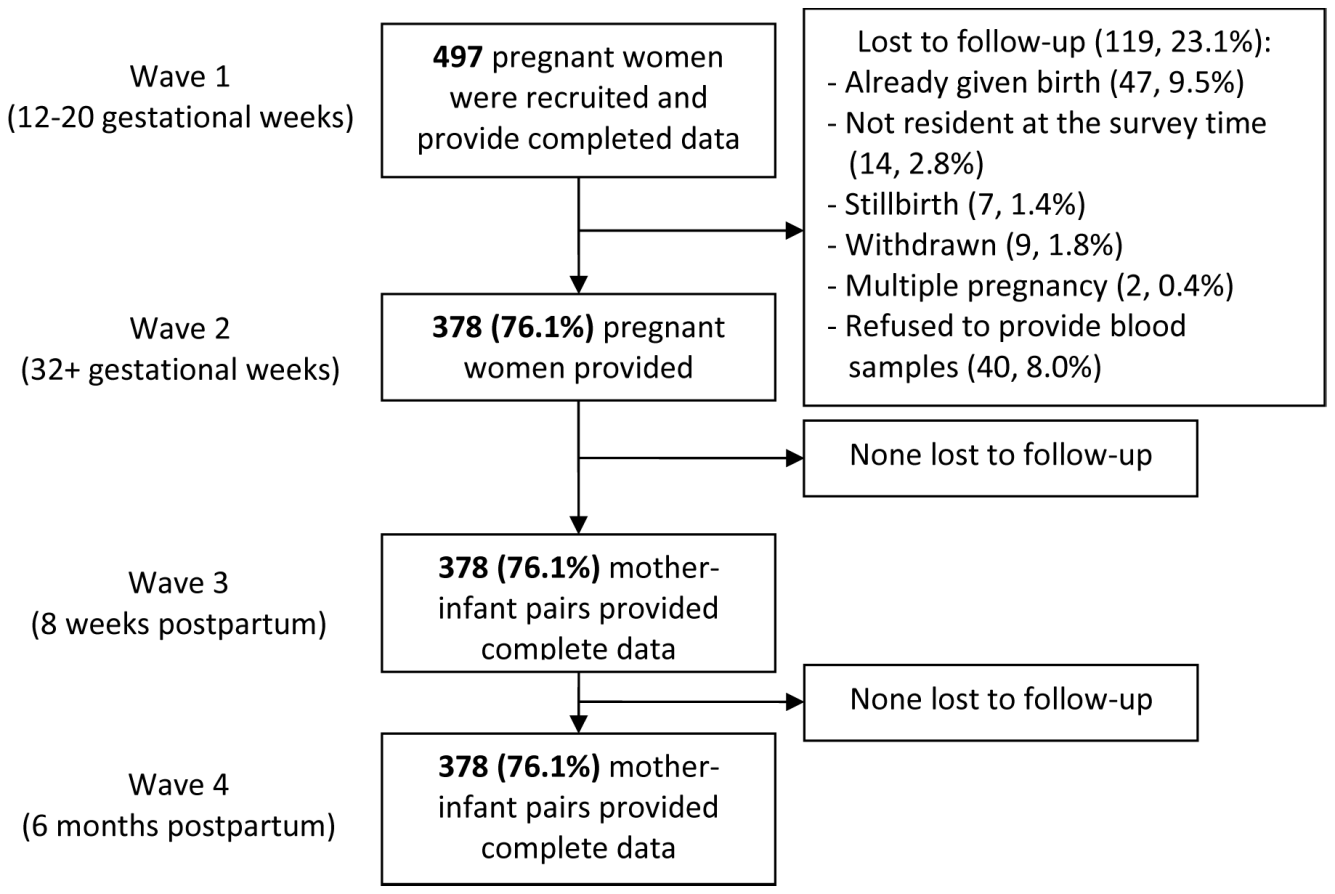
Numbers of participants and attrition by waves of data collection.

A total of 523 women were eligible to participate, of whom 497 (97%) were recruited and provided data at W1. Complete data were available for 378 mother and infant pairs and included in the analyses (see Figure 2). The demographic characteristics of the women and anthropometric indices of the infants are shown in Tables 1 and 2. There were 189/378 (50%) women with clinical symptoms of CMDs at any point of early (W1) or late pregnancy (W2) and 64/378 (16.9%) with persistent clinical symptoms of CMDs at both W1 and W2 pregnancy assessments. Overall, 59/378 (15.6%) women were experienced IDA at any antenatal study point and 6/378 (1.6%) at both points.

**Table 1:** General characteristics of the 378 pregnant women who attended four waves.

Characteristic	Values
Mother age (years), mean [SD]	26.2 [4.8]
Completed education, No. (%)	
Partial or complete primary school (Grades 1–5)	68 (18.0)
Secondary school (Grades 6–9)	202 (53.4)
High school (Grades 10–12)	46 (12.2)
Any post-secondary education	62 (16.4)
Occupation, No. (%)	
Farmer	176 (46.6)
Factory, handcraft worker or retailer	115 (30.4)
Government or private officer	46 (12.2)
Not currently engaged in income-generating activity	41 (10.8)
Gestational age or age of the infant, mean [SD]	
Wave 1 (gestation age, weeks)	16.6 [2.9]
Wave 2 (gestation age, weeks)	33.1 [2.0]
Wave 3 (infant age, weeks)	8.1 [2.0]
Wave 4 (infant age, months)	6.6 [0.5]
Number of children, No. (%)	
One	136 (36.0)
Two	169 (44.7)
Three or more	73 (19.3)
Prior miscarriages/stillbirths, No. (%)	69 (18.3)
Intimate partner physical and sexual violence, No. (%)	57 (15.1)
Intimate partner emotional abuse, controlling behavior,No. (%)	99 (26.2)
Experience of childhood abuse, No. (%)	38 (10.1)
Common mental disorders, No. (%)	
Wave 1	147 (38.9)
Wave 2	106 (28.0)
Wave 3	40 (10.8)
Wave 4	44 (12.1)
Iron deficiency anemia	
Wave 1	9 (2.4)
Wave 2	56 (14.8)
Wave 2 Urine iodine concentration (µg/L), median {IQR}	69 {41–122}
Wave 2 Urine iodine concentration <150 µg/L, No. (%)	312 (82.5)

*[SD]: Standard deviation; (%): Percentage; {IQR}: interquartile range.*

**Table 2:** Birth outcomes, health and development of 378 infants.

Characteristic	Values
Infant birthweight (gram), mean [SD]	3150 [404]
Low birthweight (<2500 grams), No. (%)	26 (6.8)
Preterm birth (<37 weeks gestation), No. (%)	53 (14.0)
Infant Weight-for-age Z score at 8 weeks, mean [SD]	−.06 [0.80]
Infant Weight-for-age Z score at 6 months, mean [SD]	−.03 [0.94]
BSID scores at 6 months, mean [SD], {range}	99.2 [12.9], {55–130}

*[SD]: Standard deviation; (%): Percentage; {range}: Min–max.*

Among the 378 women, only one participant did not initiate breastfeeding. At about 8 weeks postpartum, most mothers (318/378, 84.1%) perceived that they had sufficient breast milk to meet the infant’s needs. At 6 months postpartum, 352 (93.1%) women were still providing their infants with at least some breastmilk. The number of mothers who reported at W3 that they did not receive any support with infant care during the night was 75/378 (20.5%), during the day was 26/378 (7.0%), and with household work was 15/378 (4.1%). However at six months postpartum, these had increased to 204/378 (54.1%) at night, 81/378 (21.5%) during the day, and 123/378 (32.6%) with housework.

### Path Analysis

**Figure 3:**
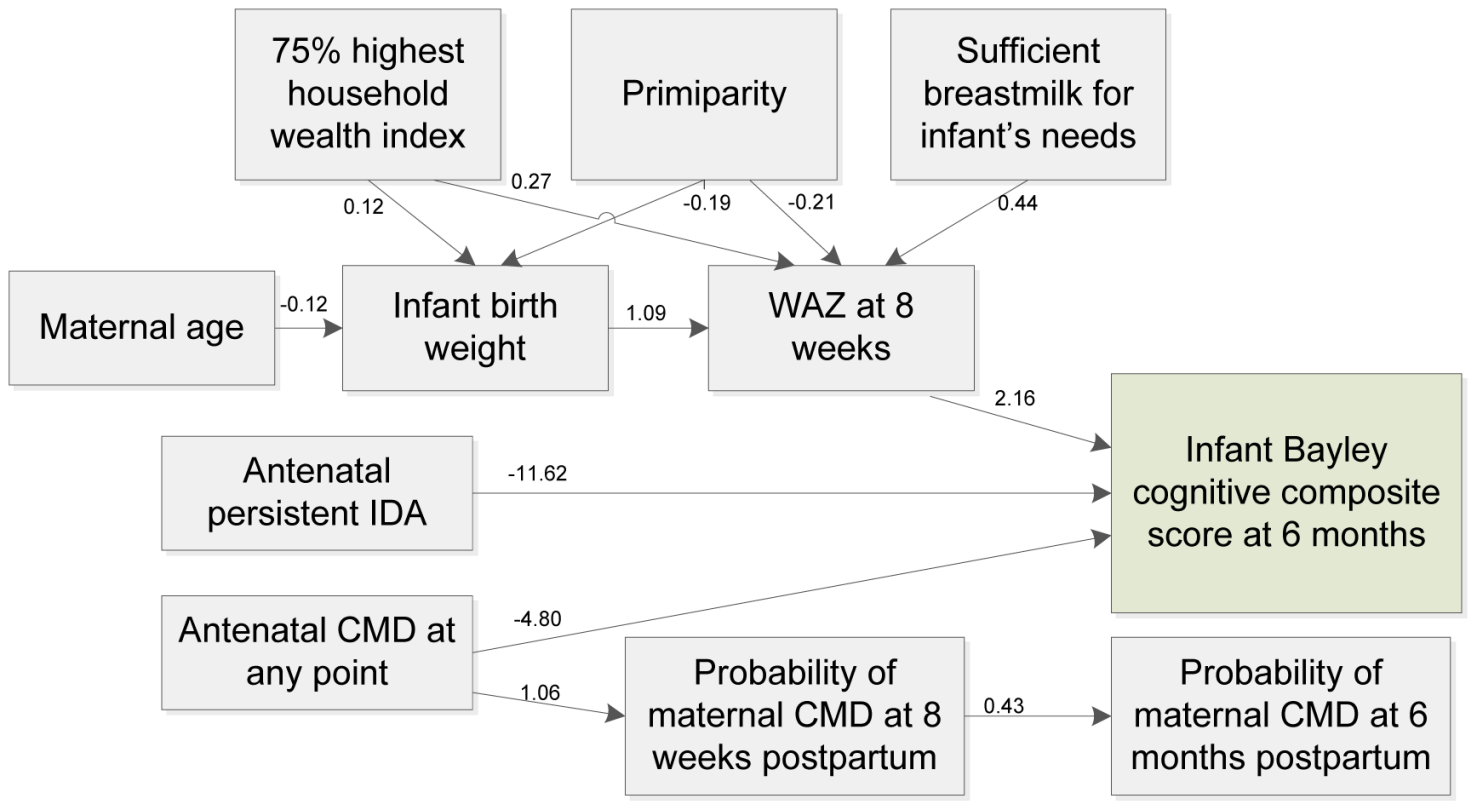
Significant pathways of the factors of infant Bayley Cognitive scores at 6 months of age. Single-headed arrows represent statistically significant directional paths (only significant paths were presented, for more details see Table 3). Path coefficients are interpreted as regression coefficients. *IDA: iron deficiency anemia; CMD: common mental disorders; WAZ: infant weight-for-age Z-score.*

Overall, there are 22 variables included in the path analysis. Details of the path model are shown in Table 3. The statistically significant paths are presented by a graphic model in Figure 3. All of the fit indices are within the range indicating that the model fits the data well.

**Table 3:** Full Path analysis model of infant Bayley Cognitive scores at 6 months of age.

Parameter estimates	Regression coefficient	95% CI
*Infant Bayley Cognitive score regressed on*		
Antenatal CMD at any point	−4.80	(−9.40; −0.20)
Antenatal Persistent IDA	−11.62	(−23.01; −0.22)
IDA at W 1 only	−3.45	(−19.13; 12.24)
IDA at W 2 only	−1.83	(−5.92; 2.25)
UIC at W2	−0.11	(−0.94; 0.34)
Infant weight-for-age Z score at W3	2.16	(0.23; 4.08)
Mother’s education level: Up to year 9	2.77	(−1.20; 6.73)
75% highest household wealth index	−3.87	(−7.62; −0.13)
Mother age >30 years	3.39	(−0.98; 7.77)
Maternal postnatal CMD at W3	1.26	(−1.02; 3.54)
Maternal postnatal CMD at W4	1.76	(−0.09; 3.62)
Maternal urine iodine concentration at W2	−0.10	(−0.30; 0.09)
Family support at W3	−1.16	(−3.39; 1.06)
History of intimate partner violence	0.01	(−1.07; 1.07)
Infant birthweight (in kilogram)	0.94	(−2.86; 4.75)
*Infant weight-for-age Z score at 8 weeks regressed on*		
Infant birthweight (in kilogram)	1.09	(0.95; 1.23)
75% highest household wealth index	0.27	(0.10; 0.44)
Sufficient breastmilk for infant’s needs	0.44	(0.21; 0.66)
Primiparity	−0.21	(−0.37; −0.04)
Mother’s education level: Up to year 9	0.05	(−0.11; 0.21)
Mother age >30 years	0.06	(−0.11; 0.22)
Antenatal CMD at any point	−0.05	(−0.19; 0.10)
Antenatal Persistent IDA	0.20	(−0.19; 0.59)
IDA at W 1 only	−0.23	(−0.85; 0.39)
IDA at W 2 only	−0.05	(−0.27; 0.16)
Family support at W3	0.06	(−0.08; 0.20)
*Infant birthweight regressed on*		
75% highest household wealth index	0.12	(0.02; 0.23)
Primiparity	−0.19	(−0.28; −0.09)
Mother age >30 years	−0.12	(−0.23; −0.02)
Mother’s education level: Up to year 9	0.02	(−0.08; 0.12)
Antenatal CMD at any point	−0.05	(−0.14; 0.04)
Antenatal Persistent IDA	0.24	(−0.16; 0.65)
IDA at W 1 only	−0.05	(−1.12; 1.03)
IDA at W 2 only	0.01	(−0.13; 0.16)
UIC at W2	−0.01	(−0.34; 0.73)
*Probability of Mother’s CMD at W3 regressed on*		
Antenatal CMD at any point	1.06	(0.34; 1.78)
Non-economic co-incidental life adversity at W3	0.87	(0.20; 1.54)
Mother’s education level: Up to year 9	−0.92	(−1.61; −0.23)
Mother age >30 years	−0.07	(−0.89; 0.76)
Primiparity	0.46	(−0.09; 1.00)
Affectionate relationship with her own mother	−0.77	(−1.54; 0.01)
Affectionate relationship with mother-in-law	0.15	(−0.36; 0.67)
History of intimate partner violence	0.05	(−0.12; 0.22)
Experience of childhood abuse	0.21	(−0.43; 0.86)
75% highest household wealth index	0.27	(−0.27; 0.80)
Family support at W3	0.19	(−0.15; 0.53)
Infant weight-for-age Z score at W3	0.04	(−0.21; 0.30)
*Probability of Mother’s CMD at W4 regressed on*		
Maternal CMD at W3	0.43	(0.13; 0.72)
Antenatal CMD at any point	0.74	(−0.17; 1.65)
Non-economic co-incidental life adversity at W4	1.87	(1.25; 2.49)
Mother’s education level: Up to year 9	0.14	(−0.62; 0.89)
Mother age >30 years	−0.21	(−0.97; 0.56)
Primiparity	0.33	(−0.4; 1.06)
Affectionate relationship with her own mother	0.43	(−0.24; 1.1)
Affectionate relationship with mother-in-law	0.28	(−0.35; 0.9)
History of intimate partner violence	−0.03	(−0.24; 0.18)
Experience of childhood abuse	0.16	(−0.56; 0.87)
75% highest household wealth index	−0.20	(−0.83; 0.43)
Family support at W4	0.10	(−0.24; 0.45)
Infant weight-for-age Z score at W4	0.18	(−0.11; 0.48)
Infant Bayley cognitive score	0.01	(−0.02; 0.02)
Fit indices	**Estimates**	
χ*^2^*/*df* (p-value)	57.5/62 (0.63)	
RMSEA (Probability RMSEA < = .05)	<0.001 (1.0)	
Comparative Fit Index	1.00	
Tucker-Lewis Index	1.00	

*W1: 12–20 weeks of gestation; W2: 32+ weeks of gestation; W3: 8 weeks postpartum; W4: 6 months postpartum.*

*RMSEA – root mean square error of approximation.*

Figure 3 shows that persistent antenatal IDA, antenatal CMD in either early or late pregnancy, and infant WAZ at 8 weeks of age had direct effects on infant BSID composite scores at 6 months of age. Infants of mothers who had persistent antenatal IDA had BSID scores 77.5% of a normative standard deviation lower than the other infants (−11.62 points; 95% CI: −23.01 to −0.22) and of those who had experienced CMD at any point during pregnancy 32% of a standard deviation cognitive score lower (−4.80 points, 95% CI: −9.40 to −0.20). Together these two risk factors contributed to more than one standard deviation reduction in infant cognitive scores at 6 months of age. Further, infants having one standard deviation of weight-for-age higher at 8 weeks old had BSID cognitive scores that were 2.16 points higher at 6 months of age (95% CI: 0.23 to 4.08).

The indirect pathways from antenatal IDA and antenatal CMD to infant cognitive development via infant birthweight, WAZ score at 8 weeks, and maternal postpartum CMD were tested simultaneously in the model. However, none of the pathways were statistically significant. There were no associations between the antenatal risk factors and infant birthweight or infant WAZ at 8 weeks of age. The link between maternal CMD during pregnancy and maternal CMD after childbirth was statistically significant. However there was no association between postpartum CMD and infant cognitive development.

Several variables had indirect associations with cognitive scores at 6 months: higher infant birthweight, higher household wealth, and maternal reports of a sufficient supply of breastmilk were indirectly positively related to the infant cognitive score. Mothers aged >30 years and who were primiparous had infants with lower cognitive scores than the others.

## Discussion

To our knowledge this is the first study to examine the effects of both iron status and common mental disorders among pregnant women living in a low-income setting on the cognitive development of their 6 month old infants. The sample was systematically recruited with high recruitment and retention fractions and all reasons for non-provision of complete data were known. The path analysis techniques used in this study determined direct and indirect effects of the probable factors on the main outcome simultaneously [39]. Our data indicate that there are direct adverse effects of persistent antenatal IDA and CMD on the cognitive development of six month-old infants. However, indirect effects of the risk factors on the outcome via infant birthweight, WAZ and maternal postpartum CMD were not found.

There is a body of evidence about the effect of maternal antenatal iron deficiency with or without anemia on adverse pregnancy outcomes including low birthweight and prematurity [40]–[44]. However, few studies have investigated the long-term effects of maternal iron deficiency during pregnancy on the neurodevelopment of the baby. To date the strongest evidence was derived from a longitudinal study of 278 American children [11], which found that low umbilical cord serum ferritin concentrations in term newborn infants correlated with impaired cognitive function at 5 year of age. However, the relationship reported in that study might actually reflect a correlation between poor placental functioning and neurodevelopmental outcome rather than a causal link between maternal IDA and child cognitive defects [45]. Our study provides evidence that maternal iron deficiency during pregnancy relates to lower infant cognitive development, through both direct and indirect potential pathways. First, low maternal iron storage could result directly in low iron transferred through the placenta to fulfill the fetus’ needs, leading to adverse consequences for child neurodevelopment [46]. Second, anemia contributes to maternal fatigue which may interfere with self-care including food preparation to ensure a sufficient dietary intake or participation in health care [21]. A limitation of our study is that it could not distinguish between these two pathways in the analyses. Antenatal persistent IDA was not prevalent in our sample (1.6%). Although point prevalence of anemia of 53% had been reported in one prior study in rural Vietnam, there were no existing data about persistent anemia/IDA on which to base our estimates, which were nevertheless higher than were found in this study [47]. We suggest that the finding of this study about the association between exposure to persistent IDA and the infant outcome should be interpreted with care. Further investigation to confirm the finding in other settings is warranted.

These data strengthen the results of previous studies about the links between maternal antenatal mental health problems and infant neurodevelopment. In the Netherlands, pregnancy-specific anxiety in mid gestation predicted low infant Bayley II Mental Development Index (Bayley II MDI) (less than percentile 25) at 8 months old (OR 1.1, 95% CI 1.02–1.18) [48], but they did not state what potential confounding factors were controlled for. Deave et al [49] reported findings from the UK Avon Longitudinal Study of Parents and Children in 9244 women and their children which showed an association between having EPDS ≥10 (usual cut-off score in high-income Anglophone countries to detect clinically significant symptoms in community samples) during pregnancy and risk of developmental delay in their infants at 18 months of age (OR 1.34, 95% CI 1.11–1.62). However, they used the Denver Developmental Screening Test to evaluate child development, which does not permit different developmental domains to be assessed. Bergman et al. [17] found prenatal stressful life events were associated with infant Bayley II MDI at 14 to 19 months old in UK (regression coefficient −3.04 (95% CI: −4.17 to −1.90). In that study, while maternal postpartum depression was controlled for, they did not control for infant nutritional status. A limited-sample-size study in 58 Canadian women who were pregnant during the ice storm observed prenatal stress exposure accounted for 11.4% of the variation in the toddlers’ Bayley II MDI at two years of age [50]. None of the existing studies in high-income countries controlled for the effect of maternal antenatal anemia or other micronutrient deficiencies. These factors may not however limit their findings because these conditions are not prevalent in the high-income settings.

Our findings are inconsistent with the conclusions of Servili et al [20] who have published the only comparable data to date in a low- or lower-middle-income country. Their Ethiopian study did not find an association between maternal antenatal mental health problems and infant cognitive functioning. There were several differences of study design that could have led to the difference with our findings. First, the study in Ethiopia did not control for IDA which we found was strongly associated with the BSID scores. The prevalence of anemia in pregnant women in Ethiopia has been reported to be as high as 29% [51]. Second, Servili et al. used raw BSID scores in the analyses, so the scores were not adjusted to account for the infant’s age. The norms of BSID change for each two weeks of infant age, therefore using raw scores introduces biases due to the age of infants. Lastly, the timing of maternal antenatal mental health assessments was only done at one time, in late pregnancy in the Ethiopian study, while our study collected these data twice: in both early and late pregnancy. Data at two points of time can show a persistent condition during pregnancy that could have a larger effect size than cross-sectional data that might detect an acute condition.

The findings of our study were also different from those of other researchers in low- and lower-middle-income countries [52]–[55] who found that postpartum CMD had an adverse effect on infant cognitive function. The reason for the difference could be that our study is the only one which takes into account antenatal CMD, a significant confounder, when examining the relationship between postpartum CMD and infant development. The mechanisms of the influence of maternal antenatal and postpartum psychological problems on child development are probably different. During pregnancy, cortisol transferring through the placenta might be the pathway [56] while parenting practices are suggested as a mediator in the postpartum period [57]. The timing of infant cognitive assessment in our study was 6 months after birth, which might not be a sufficient interval to reflect the impact of parental caregiving on the cognitive functioning of the infant. A previous study shows that the effect of parenting quality on child cognitive development could not be detected until children reached two years of age [58].

Mechanisms underlying the effects of IDA and/or CMD on infant development outcome have been discussed where else [56], [59], [60]. Both of those risk factors share the most important pathway via the elevation of maternal stress hormones (cortisol) and placental corticotropin-releasing hormone (CRH). IDA increases norepinephrine concentrations that in turn elevates the release of maternal cortisol and placental CRH [61], [62] while CMD trigger elevated maternal cortisol levels and placental CRH via the activation of maternal hypothalamic-pituitary-adrenal (HPA) axis [63]. The fetus may be exposed to maternal cortisol through the maternal-fetal blood exchange even though the placenta may protect the fetus from the effects of maternal cortisol through increasing a placental hormone, 11β-hydroxysteroid dehydrogenase type 2, which oxidizes cortisol to its inactive form, cortisone, [64], [65]. Placental CRH, which is identical to hypothalamic CRH in structure and function, is released into both mother and fetus and can act to release cortisol in the fetus [66]. Fetal adjustments to this adverse condition in utero may cause long-lasting changes in physiologic functions that render the brain vulnerable to cognitive developmental delay later in life [2]–[4], [56]. The findings of this study could not prove this mechanism, but may support it.

## Conclusions

We acknowledge several limitations. First, the loss to follow up was relatively high in this study (119/497, 23.9%). However, high rates of loss to follow-up were common in similar previous longitudinal studies including in Servilli et al. 64/258 (24.8%), Buitelaar et al. 60/230 (26.1%), Bergman et al. 240/365 (65.8%), and Laplante et al. 172/224 (76.8%). There were no significant differences in the socio-demographic, psychological, or biological characteristics between women who provided complete data and those who were not retained at the six-month postpartum follow up. The second limitation was that we used the EPDS-V to detect maternal CMD. The EPDS is a screening tool, which does not yield diagnoses and does not distinguish between depression and anxiety. However, in this setting the EPDS clinical cut-off score that we used has a high level of sensitivity (70%) and specificity (73%) when validated against a diagnostic psychiatric interview to detect CMD including depression and anxiety in perinatal women in the same setting [30]. We acknowledge that the prevalence of antenatal persistent IDA in our sample was low. In order to mitigate the effect of that limitation, we have used a robust method (WLSMV) which recommended for non-normality data to estimate the parameters of the model. Finally, even though Path Analysis is an advanced modern statistical technique it makes assumptions about linearity, additivity, normality, homoskedasticity, lack of multicollinearity, and identification. The assumptions were all checked carefully and there were very few divergences, but we acknowledge that any of these can reduce the validity of the findings. Potential confounders that were not assessed or included in the model might affect the findings [67].

Overall, we believe that the strengths of this study (a systematically recruited and representative sample and assessment of both biological and psychosocial factors) outweigh potential limitations and that the data are reliable and can be generalized with some confidence to rural Vietnam and other developing countries. They indicate that persistent IDA and CMD in pregnant women may increase the risk of lower infant cognitive development in six-month-old infants in these settings. Our study contributes to scarce literature on the relationship between maternal antenatal health and infant development in developing countries to suggest further attentions to this critical period in order to improve cognitive function of children in these settings.

## References

[1] Grantham-McGregor S, Cheung Y, Cueto S, Glewwe P, Richter L, et al (2007) Developmental potential in the first 5 years for children in developing countries. The Lancet 369: 60–70. 10.1016/S0140-6736(07)60032-4 PMC2270351 17208643

[2] deRegnier R-A, Desai S (2010) Fetal Development. In: Bremner JG, Wachs TD, editors. The Wiley-Blackwell handbook of infant development. 2nd ed. Chichester, West Sussex: Wiley-Blackwell.

[3] Barker DJ (2002) Fetal programming of coronary heart disease. Trends Endocrinol Metab 13: 364–368. 12367816 10.1016/s1043-2760(02)00689-6

[4] Desai M, Gayle D, Babu J, Ross MG (2005) Programmed obesity in intrauterine growth-restricted newborns: modulation by newborn nutrition. American Journal of Physiology Regulatory, Integrative and Comparative Physiology 288: R91–96. 10.1152/ajpregu.00340.2004 15297266

[5] Zimmermann MB, Hurrell RF (2007) Nutritional iron deficiency. Lancet 370: 511–520. 17693180 10.1016/S0140-6736(07)61235-5

[6] McLean E, Cogswell M, Egli I, Wojdyla D, de Benoist B (2009) Worldwide prevalence of anaemia, WHO Vitamin and Mineral Nutrition Information System, 1993–2005. Public Health Nutrition 12: 444–454. 18498676 10.1017/S1368980008002401

[7] Hernández-Martínez C, Canals J, Aranda N, Ribot B, Escribano J, et al (2011) Effects of iron deficiency on neonatal behavior at different stages of pregnancy. Early Human Development 87: 165–169. 21256683 10.1016/j.earlhumdev.2010.12.006

[8] Wachs TD, Pollitt E, Cueto S, Jacoby E, Creed-Kanashiro H (2005) Relation of neonatal iron status to individual variability in neonatal temperament. Developmental Psychobiology 46: 141–153. 15732057 10.1002/dev.20049

[9] Amin SB, Orlando M, Eddins A, MacDonald M, Monczynski C, et al (2010) In utero iron status and auditory neural maturation in premature infants as evaluated by auditory brainstem response. Journal of Pediatrics 156: 377–381. 19939407 10.1016/j.jpeds.2009.09.049 PMC2827634

[10] Riggins T, Miller NC, Bauer PJ, Georgieff MK, Nelson CA (2009) Electrophysiological indices of memory for temporal order in early childhood: implications for the development of recollection. Dev Sci 12: 209–219. 19143795 10.1111/j.1467-7687.2008.00757.x PMC2771175

[11] Tamura T, Goldenberg RL, Hou J, Johnston KE, Cliver SP, et al (2002) Cord serum ferritin concentrations and mental and psychomotor development of children at five years of age. Journal of Pediatrics 140: 165–170. 11865266 10.1067/mpd.2002.120688

[12] Rioux FM, Belanger-Plourde J, Leblanc CP, Vigneau F (2011) Relationship between maternal DHA and iron status and infants’ cognitive performance. Canadian Journal of Dietetic Practice and Research 72: 76. 21645426 10.3148/72.2.2011.e140

[13] Zhou SJ, Gibson RA, Crowther CA, Baghurst P, Makrides M (2006) Effect of iron supplementation during pregnancy on the intelligence quotient and behavior of children at 4 y of age: long-term follow-up of a randomized controlled trial. American Journal of Clinical Nutrition 83: 1112–1117. 16685054 10.1093/ajcn/83.5.1112

[14] Parsons AG, Zhou SJ, Spurrier NJ, Makrides M (2008) Effect of iron supplementation during pregnancy on the behaviour of children at early school age: long-term follow-up of a randomised controlled trial. British Journal of Nutrition 99: 1133–1139. 17967217 10.1017/S0007114507853359

[15] Fisher J, de Mello MC, Patel V, Rahman A, Tran T, et al (2012) Prevalence and determinants of common perinatal mental disorders in women in low- and lower-middle-income countries: a systematic review Bull World Health Organ. 90: 139–149G. 10.2471/BLT.11.091850 PMC3302553 22423165

[16] O’Connor TG, Heron J, Glover V (2002) Antenatal anxiety predicts child behavioral/emotional problems independently of postnatal depression. Journal of the American Academy of Child and Adolescent Psychiatry 41: 1470–1477. 12447034 10.1097/00004583-200212000-00019

[17] Bergman K, Sarkar P, O’Connor TG, Modi N, Glover V (2007) Maternal stress during pregnancy predicts cognitive ability and fearfulness in infancy. Journal of the American Academy of Child and Adolescent Psychiatry 46: 1454–1463. 18049295 10.1097/chi.0b013e31814a62f6

[18] Bergman K, Sarkar P, Glover V, O’Connor TG (2010) Maternal prenatal cortisol and infant cognitive development: moderation by infant-mother attachment. Biological Psychiatry 67: 1026–1032. 20188350 10.1016/j.biopsych.2010.01.002 PMC2872196

[19] DiPietro JA, Novak MF, Costigan KA, Atella LD, Reusing SP (2006) Maternal psychological distress during pregnancy in relation to child development at age two. Child Development 77: 573–587. 16686789 10.1111/j.1467-8624.2006.00891.x

[20] Servili C, Medhin G, Hanlon C, Tomlinson M, Worku B, et al (2010) Maternal common mental disorders and infant development in Ethiopia: the P-MaMiE Birth Cohort. BMC Public Health 10: 693. 21073710 10.1186/1471-2458-10-693 PMC3091583

[21] Fisher J, Tran T, La BT, Kriitmaa K, Rosenthal D, et al (2010) Common perinatal mental disorders in northern Viet Nam: community prevalence and health care use. Bull World Health Organ 88: 737–745. 20931058 10.2471/BLT.09.067066 PMC2947037

[22] National Institute of Nutrition/UNICEF (2011) A review of the nutrition situation in Viet Nam 2009–2010. Hanoi: Medical Public House.

[23] Allen LH (2000) Anemia and iron deficiency: effects on pregnancy outcome. American Journal of Clinical Nutrition 71: 1280S–1284S. 10799402 10.1093/ajcn/71.5.1280s

[24] Grote NK, Bridge JA, Gavin AR, Melville JL, Iyengar S, et al (2010) A meta-analysis of depression during pregnancy and the risk of preterm birth, low birth weight, and intrauterine growth restriction. Archives of General Psychiatry 67: 1012–1024. 20921117 10.1001/archgenpsychiatry.2010.111 PMC3025772

[25] Walker SP, Wachs T, Meeks Gardner J, Lozoff B, Wasserman G, et al (2007) Child development: risk factors for adverse outcomes in developing countries. The Lancet 369: 145–157. 10.1016/S0140-6736(07)60076-2 17223478

[26] Fisher J, Tran T, Biggs B, Tran T, Dwyer T, et al (2011) Iodine status in late pregnancy and psychosocial determinants of iodized salt use in rural northern Viet Nam. Bulletin of World Health Organization 89: 813–820. 10.2471/BLT.11.089763 PMC3209728 22084527

[27] Bayley N (2006) Bayley Scales of Infant and Toddler Development–Third Edition. San Antonio: TX: Harcourt Assessment.

[28] Bayley N (2006) Bayley Scales of Infant and Toddler Development–Third Edition: Technical Manual. San Antonio: TX: Harcourt Assessment.

[29] Cox J, Holden J, Sagovsky R (1987) Detection of postnatal depression. Development of the 10-item Edinburgh Postnatal Depression Scale. British Journal of Psychiatry 150: 782–786. 3651732 10.1192/bjp.150.6.782

[30] Tran TD, Tran T, La B, Lee D, Rosenthal D, et al (2011) Screening for perinatal common mental disorders in women in the north of Vietnam: a comparison of three psychometric instruments. Journal of Affective Disorders 133: 281–293. 21529963 10.1016/j.jad.2011.03.038

[31] Garcia-Moreno C, Jansen H, Ellsberg M, Heise L, Watts C (2005) WHO Multi-Country Study on Women’s Health and Domestic Violence Against Women: Initial Results on Prevalence, Health Outcomes and Women’s Reponses. Geneva: World Health Organization.

[32] Ellsberg M, Jansen HA, Heise L, Watts CH, Garcia-Moreno C (2008) Intimate partner violence and women’s physical and mental health in the WHO multi-country study on women’s health and domestic violence: an observational study. Lancet 371: 1165–1172. 18395577 10.1016/S0140-6736(08)60522-X

[33] Tran T (2004) Community based evidence about the health care system in rural Viet Nam. NewcastleAustralia: University of Newcastle. 297 p.

[34] WHO/UNICEF/ICCIDD (2001) Assessment of Iodine Deficiency Disorders and Monitoring their Elimination: A Guide for Programme Managers, WHO/NHD/01.1 2nd ed. Geneva: World Health Organization.

[35] Laungani P (2000) Postnatal depression across cultures:conceptual and methodological considerations. International Journal of Health Promotion and Education 38: 86–94.

[36] Bayley N (2006) Bayley Scales of Infant and Toddler Development–Third Edition: Administration Manual. San Antonio: TX: Harcourt Assessment.

[37] WHO/UNICEF/UNU (2001) Iron deficiency anaemia, assessment, prevention and control: a guide for programme managers. Geneva: World Health Organization.

[38] Kline RB, Ebooks Corporation. (2011) Principles and practice of structural equation modeling. Methodology in the social sciences. 3rd ed. New York: Guilford Press. pp. xvi, 427 p.

[39] Pearl J (2009) Causality: models, reasoning, and inference; 2nd, editor. New York: Cambridge Univ Press.

[40] Kidanto HL, Mogren I, Lindmark G, Massawe S, Nystrom L (2009) Risks for preterm delivery and low birth weight are independently increased by severity of maternal anaemia. South African Medical Journal 99: 98–102. 19418670

[41] Jaleel R, Khan A (2008) Severe anaemia and adverse pregnancy outcome. Journal of Surgery Pakistan (International) 13: 147–150.

[42] Marahatta R (2007) Study of anaemia in pregnancy and its outcome in Nepal Medical College Teaching Hospital, Kathmandu, Nepal. Nepal Med Coll J 9: 270–274. 18298019

[43] Geelhoed D, Agadzi F, Visser L, Ablordeppey E, Asare K, et al (2006) Maternal and fetal outcome after severe anemia in pregnancy in rural Ghana. Acta Obstetricia et Gynecologica Scandinavica 85: 49–55. 16521680 10.1080/00016340500334794

[44] Lone FW, Qureshi RN, Emanuel F (2004) Maternal anaemia and its impact on perinatal outcome. Tropical Medicine and International Health 9: 486–490. 15078267 10.1111/j.1365-3156.2004.01222.x

[45] Fleming RE (2002) Cord serum ferritin levels, fetal iron status, and neurodevelopmental outcomes: correlations and confounding variables. Journal of Pediatrics 140: 145–148. 11865262 10.1067/mpd.2002.121931

[46] Lozoff B, Beard J, Connor J, Barbara F, Georgieff M, et al.. (2006) Long-lasting neural and behavioral effects of iron deficiency in infancy. Nutrition Reviews 64: S34–43; discussion S72–91. 10.1301/nr.2006.may.S34-S43 PMC1540447 16770951

[47] Trinh L, Dibley M, Byles J (2007) Antenatal care procedures and information reported by women in three rural areas of Vietnam. Southeast Asian Journal of Tropical Medicine and Public Health 38: 927–935. 18041314

[48] Buitelaar JK, Huizink AC, Mulder EJ, de Medina PG, Visser GH (2003) Prenatal stress and cognitive development and temperament in infants. Neurobiology of Aging 24 Suppl 1: S53–60; discussion S67–58. 10.1016/s0197-4580(03)00050-2 12829109

[49] Deave T, Heron J, Evans J, Emond A (2008) The impact of maternal depression in pregnancy on early child development. BJOG 115: 1043–1051. 18651886 10.1111/j.1471-0528.2008.01752.x

[50] Laplante DP, Barr RG, Brunet A, Galbaud du Fort G, Meaney ML, et al (2004) Stress during pregnancy affects general intellectual and language functioning in human toddlers. Pediatric Research 56: 400–410. 15240860 10.1203/01.PDR.0000136281.34035.44

[51] Gibson RS, Abebe Y, Stabler S, Allen RH, Westcott JE, et al (2008) Zinc, gravida, infection, and iron, but not vitamin B-12 or folate status, predict hemoglobin during pregnancy in Southern Ethiopia. The Journal of nutrition 138: 581–586. 18287370 10.1093/jn/138.3.581 PMC2440679

[52] Patel V, Desouza N, Rodrigues M (2003) Postnatal depression and infant growth and development in low income countries: A cohort study from Goa, India. Archives of Disease in Childhood 88: 34–37. 12495957 10.1136/adc.88.1.34 PMC1719257

[53] Black MM, Baqui AH, Zaman K, McNary SW, Le K, et al (2007) Depressive symptoms among rural Bangladeshi mothers: implications for infant development. Journal of Child Psychology and Psychiatry and Allied Disciplines 48: 764–772. 10.1111/j.1469-7610.2007.01752.x 17683448

[54] Hadley C, Tegegn A, Tessema F, Asefa M, Galea S (2008) Parental symptoms of common mental disorders and children’s social, motor, and language development in sub-Saharan Africa. Annals of Human Biology 35: 259–275. 18568592 10.1080/03014460802043624

[55] Galler J, Harrison R, Ramswy F, Forde V, Butler S (2000) Maternal depressive symptoms affect infant cognitive development in Barbados. Journal Child Psychology and Psychiatry 41: 747–757. 11039687

[56] Talge NM, Neal C, Glover V (2007) Antenatal maternal stress and long-term effects on child neurodevelopment: how and why? Journal of Child Psychology and Psychiatry and Allied Disciplines 48: 245–261. 10.1111/j.1469-7610.2006.01714.x PMC11016282 17355398

[57] Murray L, Halligan S, Cooper P (2010) Effects of postnatal depression on mother-infant interactions and child development. In: Bremner JG, Wachs TD, editors. Wiley-Blackwell handbooks of developmental psychology. 2nd ed. Chichester, West Sussex: Wiley-Blackwell.

[58] Lugo-Gil J, Tamis-LeMonda CS (2008) Family resources and parenting quality: Links to children’s cognitive development across the first 3 years. Child development 79: 1065–1085. 18717907 10.1111/j.1467-8624.2008.01176.x

[59] Field T (2011) Prenatal depression effects on early development: a review. Infant Behav Dev 34: 1–14. 20970195 10.1016/j.infbeh.2010.09.008

[60] Allen LH (2001) Biological mechanisms that might underlie iron’s effects on fetal growth and preterm birth. J Nutr 131: 581S–589S. 11160591 10.1093/jn/131.2.581S

[61] Dallman PR (1986) Biochemical basis for the manifestations of iron deficiency. Annu Rev Nutr 6: 13–40. 3524613 10.1146/annurev.nu.06.070186.000305

[62] Gulmezoglu AM, Mahomed K, Hofmeyr GJ, Nikodem VC, Kramer T (1996) Fetal and maternal catecholamine levels at delivery. J Perinat Med 24: 687–691. 9120753 10.1515/jpme.1996.24.6.687

[63] Wadhwa PD (2005) Psychoneuroendocrine processes in human pregnancy influence fetal development and health. Psychoneuroendocrinology 30: 724–743. 15919579 10.1016/j.psyneuen.2005.02.004

[64] Mulder EJ, Robles de Medina PG, Huizink AC, Van den Bergh BR, Buitelaar JK, et al (2002) Prenatal maternal stress: effects on pregnancy and the (unborn) child. Early Hum Dev 70: 3–14. 12441200 10.1016/s0378-3782(02)00075-0

[65] Brown RW, Diaz R, Robson AC, Kotelevtsev YV, Mullins JJ, et al (1996) The ontogeny of 11 beta-hydroxysteroid dehydrogenase type 2 and mineralocorticoid receptor gene expression reveal intricate control of glucocorticoid action in development. Endocrinology 137: 794–797. 8593833 10.1210/endo.137.2.8593833

[66] Petraglia F, Florio P, Nappi C, Genazzani AR (1996) Peptide signaling in human placenta and membranes: autocrine, paracrine, and endocrine mechanisms. Endocrine Reviews 17: 156–186. 8706630 10.1210/edrv-17-2-156

[67] VanderWeele TJ (2012) Invited commentary: structural equation models and epidemiologic analysis. American Journal of Epidemiology 176: 608–612. 22956513 10.1093/aje/kws213 PMC3530375

